# Medical Image Segmentation with Learning Semantic and Global Contextual Representation

**DOI:** 10.3390/diagnostics12071548

**Published:** 2022-06-25

**Authors:** Mohammad D. Alahmadi

**Affiliations:** Department of Software Engineering, College of Computer Science and Engineering, University of Jeddah, Jeddah 23890, Saudi Arabia; mdalahmadi@uj.edu.sa

**Keywords:** attention mechanism, medical images, medical segmentation

## Abstract

Automatic medical image segmentation is an essential step toward accurate diseases diagnosis and designing a follow-up treatment. This assistive method facilitates the cancer detection process and provides a benchmark to highlight the affected area. The U-Net model has become the standard design choice. Although the symmetrical structure of the U-Net model enables this network to encode rich semantic representation, the intrinsic locality of the CNN layers limits this network’s capability in modeling long-range contextual dependency. On the other hand, sequence to sequence Transformer models with a multi-head attention mechanism can enable them to effectively model global contextual dependency. However, the lack of low-level information stemming from the Transformer architecture limits its performance for capturing local representation. In this paper, we propose a two parallel encoder model, where in the first path the CNN module captures the local semantic representation whereas the second path deploys a Transformer module to extract the long-range contextual representation. Next, by adaptively fusing these two feature maps, we encode both representations into a single representative tensor to be further processed by the decoder block. An experimental study demonstrates that our design can provide rich and generic representation features which are highly efficient for a fine-grained semantic segmentation task.

## 1. Introduction

Medical images have been used in the diagnosis of various diseases in the field of health and medicine. Today, these images are typically analyzed by Computer-Aided Diagnosis (CAD) systems. More specifically, doctors and pathologists utilize CAD systems to precisely interpret medical images to make an accurate diagnosis and apply the appropriate treatment method to patients [1,2]. Several advantages of using these CAD systems include reducing the cost, time, and error of humans in analyzing medical images. CAD systems are used to perform tasks that include image segmentation, classification, and detection. Medical image segmentation methods seek to divide images into meaningfully different areas, such as disease-prone and healthy regions, so that medical professionals can focus on disease areas with great precision. Yet, segmentation of medical images is a challenging task due to some factors such as inherent noise value in these images, low contrast, the presence of multiple similar tissues, lesion sizes, color shift, complex geometry, and non-uniform lighting system between different laboratories.

**Figure 1:**
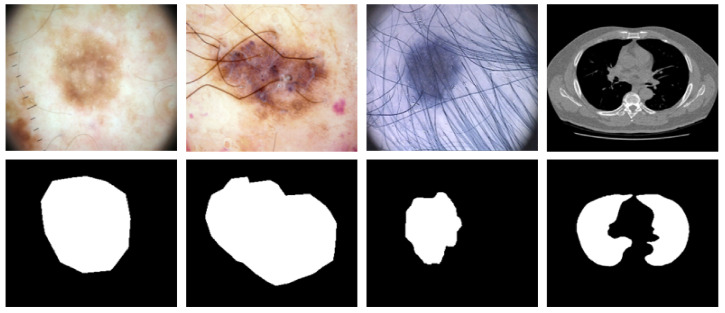
Some examples of medical images with their corresponding segmentation images. The three left images show the skin lesion segmentation whereas the right one indicates the segmentation of lung nodule.

Medical image segmentation has a wide range of applications, such as the segmentation of skin lesions and lung cancer. Skin lesion segmentation separates areas of skin that are likely to be infected by cancer from healthy areas. In such applications, early diagnosis of the disease is crucial because the disease can be treated in the early stages and prevented from spreading to other parts of the body. According to the study [3], if such diseases, which are caused by the unusual growth of melanocytes, are diagnosed early, the five-year relative survival rate becomes 92%. Lung cancer is one of the most dangerous types of cancer, which kills many people worldwide every year. According to statistics [4], the mortality rate of this cancer is 40%, and it causes the death of more than one million people annually [4]. Figure 1 shows examples of medical images and the corresponding segmentation maps.

Although Convolutional Neural Network (CNN) methods are highly effective in segmentation tasks, they are not capable of effectively modeling long-range semantic dependencies caused by the characteristics of the convolutional operations and the restricted receptive field size in convolution layers, even when the dilated/atrous sampling techniques are utilized [5]. These deficiencies reduce network performance, especially in dealing with images that have complex structures, such as highly detailed medical images with similar textures. To address the problem of the restricted receptive field of typical CNNs, several studies have been conducted [5,6,7,8,9,10,11,12]. Among the proposed methods, Transformer-based architectures that utilize the self-attention mechanism have achieved the highest ability to model long-range semantic dependencies and global contexts. Recently, several studies have been performed to adapt Transformers to image recognition applications [13,14], especially in the field of medical image segmentation [15,16,17]. All the aforementioned methods lack a distinctive mechanism to adaptively integrate the local and global contextual representations. More specifically, these methods can model long-range semantic dependencies and global contexts well but they perform more weakly in local information modeling than CNN models. Therefore, a mechanism is needed to model the global contextual features derived from the Transformer module along with the local semantic CNN representation.

In this paper, we present a two-stream pipeline network to tackle the limitation of the state-of-the-art (SOAT) methods. First, we extract local semantic information using a CNN module. Next, we employ a Transformer module to model long-range contextual representations.

Unlike the proposed approach by [15], which merely concatenates the local and global features, our proposed model adaptively fuses these feature maps and highlights the important regions by employing a spatial attention module. Our empirical findings from extensive experiments confirm that the proposed method not only provides a strong semantic segmentation map but can also pay more attention to the overlapped boundary area. The key contributions of the paper are as follows:

- Using Transformer model on the network bottleneck to generate a complementary representation for the CNN features;
- Proposing spatial attention mechanism to adaptively scale the important regions inside the given feature map;
- End-to-end design for coupling CNN and Transformer models.

The remainder of this paper is organized as follows: Section 2 presents the related work in more detail, and the proposed method and the experimental results are discussed in Section 3 and Section 4, respectively. Finally, Section 5 presents the conclusion.

## 2. Related Work

### 2.1. Handcrafted Approaches

Handcrafted feature-based methods utilize the information present in the image itself and are typically used by traditional machine learning approaches, such as Support Vector Machines, for computer vision tasks. Several handcrafted feature-based approaches have been proposed in the medical image segmentation domain that use techniques such as histogram thresholding methods [18,19,20], unsupervised color-based methods [21,22,23], region-merging-based approaches [24,25,26], active contour methods [27,28,29], and morphological operations-based methods [30,31]. In retina blood vessel segmentation applications, Zhang et al. [32] applied denoising, normalization, and eliminating artifacts in the retina images and utilized mathematical morphology operation to segment the input images. Furthermore, using the segmentation results, they employed a binary random forest classifier to classify the images into lesion and non-lesion areas. Fraz et al. [33] observed the shift in a branching pattern, diameter, and tortuosity of retinal blood vessel morphology, to segment blood vessels in retinal images. Lam et al. [34] proposed a multi-concavity to segment healthy and unhealthy pixels in retinal images. The authors employed differentiable concavity measures to take bright lesions in the input images.

In skin lesion segmentation applications, Riaz et al. [27] proposed an active contours-based method to segment melanoma areas in dermoscopy images calculating the Kullback–Leibler divergence between the skin and lesion. Then, they used image local binary patterns features to extract the periphery of the melanoma area. Pereira et al. [19] utilized a histogram and clustering-based approach for skin lesion segmentation. They found an optimal region of interest (ROI) according to a medium between the ROI with the highest gradient in the orthogonal direction of their boundary line, and another ROI with a smaller gradient and larger area. Ashour et al. [22] addressed the skin lesion segmentation problem by proposing a genetic algorithm (GA) based approach which reduces the indeterminacy of the input dermoscopy images by using the neutrosophic set (NS) operation. Then, they applied the k-means clustering algorithm to segment the skin lesion regions. In lung segmentation applications, Hu et al. [35] proposed an approach to identify the lungs in pulmonary X-ray CT images as follows. First, they used a gray-level thresholding technique to extract the lung region from the CT images. Then, they identified the anterior and posterior junctions of the lungs to separate the left and right lungs. Finally, the segmentation result was obtained by applying a sequence of morphological operations that smooth the irregular boundaries. In another study, Mansoor et al. [36] presented a two-steps method for pathological lung image segmentation as follows. First, they utilized a fuzzy connectedness (FC) algorithm to conduct initial lung parenchyma extraction alongside using rib-cage information to estimate the lung volume that compares the volume differences between the rib cage and FC. Next, they identified the abnormal imaging patterns that might have been omitted during the foremost stage of the algorithm by employing texture-based features.

Although several handcrafted feature-based approaches have been proposed to tackle the medical images segmentation problem, they extract features heuristically and therefore they do not produce accurate results. More specifically, they typically fail in situations where there are problems such as fuzzy lesion borders, the presence of multiple tissues that are similar, hair artifacts, low contrast, and patient-specific properties that may change tissue colors.

### 2.2. Deep Learning Approaches

Deep learning approaches have grown rapidly and now they are the most prominent methods for medical images segmentation. Fully Convolutional Neural Network (FCN) [37] is one of the first methods introduced for image segmentation, which works based on the deep convolutional and deconvolution layers. In these networks, the weight of the kernels used for convolution operations is learned by the network, and, after proper model training, these networks are able to extract discriminative features to segment input images. U-Net [38] is an extended idea from the FCN for medical image semantic segmentation applications. The U-Net architecture is designed symmetrically U-shaped and consists of two main paths: the encoder path, which is responsible for reducing the dimensionality of the input images and extracting feature maps, and the decoder path, which is responsible for producing the segmentation map by applying series of up-convolutional layers. This architecture also utilizes a series of skip connections for integrating deep and shallow features acquired from encoder and decoder paths at different scales. Other successful CNN-based architectures, such as 3D U-Net [39], Unet++ [40], SegNet [41], hourglass [42] and DeepLab [5], have also been introduced in recent years and are used in several medical image segmentation applications. Some recent CNN-based approaches are reviewed in the following.

Liu et al. [43] utilized edge prediction-based auxiliary information to segment lesion areas in dermoscopic images. The proposed method employs a cross-connection layer module and creates multi-scale features to improve the network performance. Tong et al. [44] extended the original U-Net model by adding a triple attention mechanism. The first attention module computes contextual information to select regions. The second and third attention modules apply spatial and channel attention to catch correlation between features. This triple attention mechanism allows the network to concentrate on more relevant regions. Kim et al. [45] proposed a four-region segmentation technique to separate different parts of the lung in chest X-ray images and apply an ensemble strategy with five diverse models to quantify COVID-19 pneumonia.

CNN-based methods are favorably efficient in segmentation tasks but are not able to effectively model long-range semantic dependencies. Several methods have been introduced to address this problem, among which Transformer-based architectures have been the most efficient. These models use the self-attention mechanism and are highly capable of modeling the long-range semantic dependencies and global contexts. Liu et al. [46] used a two patch-based strategies for medical image segmentation based on a vision transformer. Mode specifically, they used a patch-based contrastive module to improve the feature representation by enforcing locality conditions. Moreover, they eliminated artifacts from the patch splitting by employing a 3D window/shifted-window multi-head self-attention module. Meng et al. [47] utilized the global information of CT images to recognize morphologic margins of liver tumors and used a multi-scale feature fusion network segmenting tumor areas. Xu et al. [48] combined a Transformer module as an encoder into the U-Net model to balance the accuracy and efficiency of the Transformer block. Furthermore, using special skip-connections, they passed all multi-scale feature maps, created in transformer and convolutional blocks, into the decode to integrate the spatial information of the input data into the model.

The main limitation of the CNN networks is the ability to capture the global contextual representation as it is only capable of modeling the local representation. On the other hand, Transformer unlike the CNN models are highly capable of capturing the long-range connectively but less effective in reconstructing local information. To benefit from both architecture designs, we combine these two networks with an extra attention mechanism to perform a fine-grained semantic segmentation task. In the next section, we present our proposed method in a comprehensive manner.

## 3. The Proposed Model

**Figure 2:**
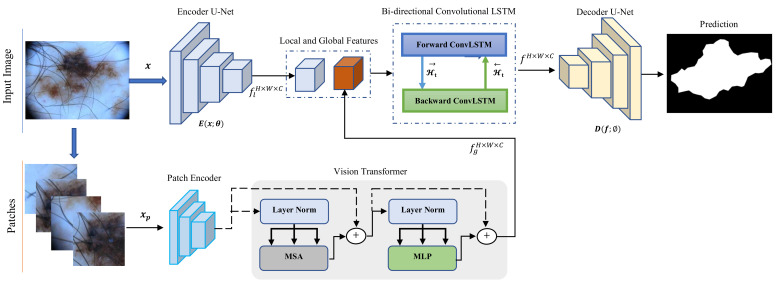
The structure of our proposed network for segmenting medical images. Our design offers a two parallel mechanism to capture both local semantic and global contextual representation, which are further fused using the ConvLSTM module.

Transformer architecture is designed in such a way that patch-wise training is faster than the case of feeding the entire image into the network. However, in a patch-wise training strategy, the network cannot learn information or dependencies for inter-patch pixels. This strategy is not a suitable mechanism for medical image segmentation tasks due to the fact that in medical images there are semantic dependencies between different pixels of images. To address this issue, we proposed a two-branches network including a Transformer branch that analyzes image patches and a CNN branch that operates on the original resolution of the input image. This two-branches structure increases the network’s overall understanding of the images by effectively distilling local semantic information derived from the CNN module and the long-range contextual representation of the Transformer model. Figure 2 depicts the architecture of our suggested hybrid network.

The Transformer branch divides each input image into 16 patches of size I/4×I/4, where the dimension of the original image is denoted by *I*, and fed each patch to the network. Next, based on the location of each patch, the output feature maps are re-sampled to produce the output feature maps. Furthermore, in the CNN branch, a seminal U-Net encoder is incorporated to model the local semantic representation. Given that the CNN branch emphasizes more delicate details and the Transformer branch concentrates on high-level information, our approach improves the network’s performance. To further effectively combine these two feature maps, we proposed to include the bi-directional ConvLSTM module in the bottleneck of the network to adaptively combine and generate the aggregated feature map for the decoding path. We argue that the suggested architecture is capable of learning both local and global characteristics of the input image which is critical for the segmentation task. In the next subsections, we explain each part in more detail.

### 3.1. Local Semantic Representation

The first branch of the proposed method utilizes the CNN encoder to capture local semantic representation. The local feature extracted by the CNN module contains rich and generic information for modeling semantic dependency among local pixels, which is crucial for the segmentation task. To this end, we consider the input image *x*, CNN encoder module *E* parametrized with θ to produce the semantic representation:

(1) $$e=E\left(\theta ;x\right).$$

In our design, the CNN encoder module can follow any well-known structure, hence, we utilize the Xception encoder [49] to produce better fine-grained representation. The Xception model was initially proposed for the object classification task and exhibited excellent performance on several challenging benchmarks. It is further utilized for the segmentation task and the tremendous achievement obtained by this network. Due to the nature of the inception module incorporated in this CNN structure, it is an ideal network for multi-scale object description. With all these characteristics along with the literature report on the advances of the Xception model for better feature representation, we utilized this as an encoder of our network.

### 3.2. Global Contextual Representation

To predict the pixel-wise label of an image $x\in R^{H\times W\times C}$, with *C* as the number of channels and a spatial resolution of H×W, we first split the *x* shape into a series of flattened 2D patches $x_{p}^{i}\in R\left(i=1,\ldots ,N\right)$, where each input image will have $N=\left(H\times W\right)/P^{2}$ number of patches of size P×P. Then, we used a linear projection vector to get a latent D-dimensional embedding space from the vectorized patches $x_{p}$. Using the below patch embedding equation, we are assured that the positional information is present.

(2) $$z_{0}=\left[x_{p}^{1}E;x_{p}^{2}E;\cdots ;x_{p}^{N},E\right]+E_{pos}$$

where the patch embedding projection is represented by $E\in R^{\left(P^{2}C\right)}\times D$ and the position embedding is indicated by $E_{\mathrm{pos}\;}\in R^{N\times D}$.

After we achieved the embedding space, in the form of a layer, we feed forward it through a multi-scale context block, made up of multi-headed self-attention (MSA), and a stack of transformer blocks, made up of multi-layer perceptron (MLP) layers [13]. Equations (3) and (4) depict these two blocks.

(3) $$z_{i}^{\prime }=\mathrm{MSA}\left(\mathrm{Norm}\left(z_{i-1}\right)\right)+z_{i-1}$$

(4) $$z_{i}=\mathrm{MLP}\left(\mathrm{Norm}\left(z_{i}^{\prime }\right)\right)+z_{i}^{\prime },$$

where the layer normalization is denoted by Norm and the individual block is represented by *i*. The MLP consists of two linear layers and the MSA block consists of *n* parallel self-attention (SA) heads. The transformer module produces a global contextual representation corresponding to each patch. To reconstruct the image level representation, using the location of each patch, we resample the output feature maps to produce the image level representation.

### 3.3. ConvLSTM Module

Standard LSTM uses full connections in state-to-state and input-to-state transitions. This means that these methods do not consider spatial correlation, which is the central limitation of this method. Shi et al. proposed ConvLSTM [50] to address this problem. The ConvLSTM uses convolution operations in transferring input-to-state and state-to-state. From a mathematical aspect, the ConvLSTM comprises three controlling gates: an input gate $i_{t}$, an output gate $o_{t}$, and a forget gate $f_{t}$ to access, update, and clear memory cell $C_{t}$. We formally define the formula that models ConvLSTM as follows:

(5) $$\begin{array}{cc} i_{t} & =\sigma \left(W_{xi}*X_{t}+W_{hi}*H_{t-1}+W_{ci}*C_{t-1}+b_{i}\right)\\ f_{t} & =\sigma \left(W_{xf}*X_{t}+W_{hf}*H_{t-1}+W_{cf}*C_{t-1}+b_{f}\right)\\ C_{t} & =f_{t}\circ C_{t-1}+i_{t}\tanh\left(W_{xc}*X_{t}+W_{hc}*H_{t-1}+b_{c}\right)\\ o_{t} & =\sigma \left(W_{xo}*X_{t}+W_{ho}*H_{t-1}+W_{co}\circ C_{t}+b_{c}\right)\\ H_{t} & =o_{t}\circ \tanh\left(C_{t}\right), \end{array}$$

where ∘ and * mark Hadamard function and convolutional operation, respectively. $X_{t}$ states the input tensor, $H_{t}$ notes the hidden state tensor, $C_{t}$ shows the memory cell tensor, $W_{x*}$ marks an input state 2D Convolution kernel, and $W_{h*}$ notes a hidden state 2D Convolution kernel. $b_{i}$, $b_{f}$, $b_{o}$, and $b_{c}$ show the bias terms.

In our architecture, we employed BConvLSTM [51] as it uses recalibrated feature pyramid encoder that maps the features to a single multi-scale representation. More specifically, BConvLSTM comprises two ConvLSTMs, one used to process input data in the forward path and the other to process data in the backward path direction. A standard ConvLSTM merely processes forward-direction dependencies, whereas BConvLSTM decides on the current input concerning the data dependencies in both directions. A study by Cui et al. [52] have shown that considering both forward and backward temporal perspectives improve the predictive performance of the model. We can consider the BConvLSTM as a two separate standard ConvLSTMs: therefore, we need two sets of parameters for backward and forward states. BConvLSTM output can be modeled as follows:

(6) $$Y_{t}=\tanh\left(W_{y}^{\vec{H}}*{\vec{H}}_{t}+W_{y}^{\overset{\leftarrow }{H}}{\overset{\leftarrow }{H}}_{t}+b\right),$$

where the forward hidden state tensors are denoted by $H_{t}$, the backward hidden state tensors are indicated by $H_{t}$, the final Spatio-temporal information-based output is marked by $Y_{t}\in R^{F_{l}\times W_{l}\times H_{l}}$, and the bias term is shown by *b*. Furthermore, we used the hyperbolic tangent tanh to integrate in a non-linear way the output of both the forward and backward states.

### 3.4. Decoder

The last module incorporated in our design is the CNN decoding block. Our decoder follows the regular U-Net decoder with five deconvolutional blocks to gradually decode and upsample the encoded feature to the segmentation map.

## 4. Experimental Results

We evaluated our proposed method on different datasets with different applications. Initially, we used three datasets, ISIC 2017 [53], ISIC 2018 [54] and PH^2^ [55], to report the performance of the proposed method in the skin lesion segmentation task. Then, we used the lung dataset to evaluate the performance of the proposed method on the lung area segmentation task. For the implementation, we trained the network from scratch for all datasets using the PyTorch framework in the Python V3 programming language. Our experiments were performed on the same machine, with NVIDIA GTX 3090 GPU and a batch size of eight without any data augmentation. We utilized the Adam optimizer and set an initial learning rate of $1\times 10^{-3}$ and a decay rate of $1\times 10^{-4}$ for 100 epochs to train the network. We terminated the model training process when the validation does not change in 10 consecutive epochs. For having a stable starting point for the network, we used a standard normal distribution for initializing the model weights.

In the following, we describe the metrics we used to evaluate our model’s performance. Additionally, the specifications of the datasets used and the results obtained in the model evaluation stage for each of these datasets are explained. Furthermore, the performance of the proposed model on each of the datasets has been compared with other state-of-the-art methods in the literature.

### 4.1. Evaluation Metrics

In order to evaluate our proposed model’s performance from different aspects, we have used the known metrics of accuracy (ACC), specificity (SP), sensitivity (SE), and Dice (DSC) score. Each of these metrics examines the specific capabilities of the proposed model. In the following, we first explain the concepts needed to sense the metrics, and then we illustrate the calculation formula of these metrics.

The True-Positive (TP) indicates a result where the trained model correctly predicts a non-healthy tissue pixel in the input image. The False-Positive (FP) indicates a result where the trained model falsely predicts a non-healthy tissue pixel in the input image. The True-Negative (TN) indicates a result where the trained model correctly predicts a healthy tissue pixel in the input image. The False-Negative (FN) indicates a result where the trained model falsely predicts a healthy tissue pixel in the input image.

**Accuracy** implies the percentage of correct prediction,

(7) $$\mathrm{ACC}=\frac{\mathrm{TP}+\mathrm{TN}}{\mathrm{TP}+\mathrm{TN}+\mathrm{FP}+\mathrm{FN}};$$

**Specificity** implies the proportion of FP that are correctly identified by model,

(8) $$\;\mathrm{Specificity}\;=\frac{\mathrm{TN}}{\mathrm{TN}+\mathrm{FP}};$$

**Sensitivity** denotes the proportion of predicted TP that are correctly identified by model,

(9) $$\;\mathrm{Sensitivity}/\;\mathrm{Recall}\;=\frac{\mathrm{TP}}{\mathrm{TP}+\mathrm{FN}};$$

**F1 score**, also known as Dice Score (DSC), is a weighted average of the precision and recall,

(10) $$\;\mathrm{DSC}\;\mathrm{score}\;=\frac{2\times \mathrm{TP}}{2\times \mathrm{TP}+\mathrm{FP}+\mathrm{FN}}.$$

### 4.2. Datasets

#### 4.2.1. ISIC 2017 Dataset

One of the most well-known datasets in the field of dermoscopic images segmentation for skin cancer diagnosis is the International Skin Imaging Collaboration (ISIC) 2017. Researchers have gathered this dataset by taking 2000 dermoscopic image samples using a skin-surface reflection elimination technique that captures images of the skin surface in deep detail [53]. To prepare this dataset for skin lesion segmentation, lesion localization, and skin disease classification tasks, each of the image samples has been annotated by clinical experts using a semi-automated or manual process. The purpose of this research is image segmentation. For this purpose, similar to the research conducted in [56], we first randomly separated the dataset into three sets: training set, validation set, and testing set, each of which contains 1250, 150, and 600 images, respectively. Besides, to reduce network load and speed up the network training process, we resized all image’s spatial dimensions to 256 × 256 pixels in the pre-processing stage.

**Figure 3:**
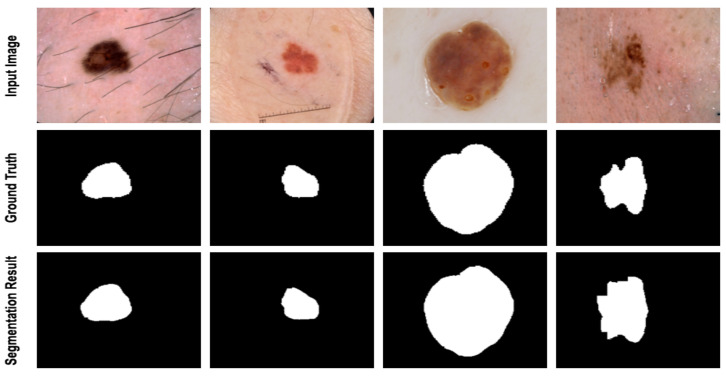
Segmentation results of the proposed method on ISIC 2017. The proposed method produces a smooth segmentation result on the boundary area and separates the lesion area from the overlapped background.

Table 1 depicts the evaluation results of our proposed model on the ISIC 2017 dataset. The results illustrated that the proposed method outperformed the state-of-the-art approaches in all metrics, except MCGU-NET using sensitivity metric. Some of the results obtained from the semantic segmentation of the proposed method on the ISIC 2017 dataset are shown in Figure 3. The segmentation results illustrate that our model accurately separates the lesion area from the healthy parts of the skin.

#### 4.2.2. ISIC 2018 Dataset

To conduct further research on the tasks of skin lesion segmentation, lesion localization and skin disease classification and to improve melanoma diagnosis, the ISIC 2018 database [54] has been created by an international collaboration. This dataset comprises 2594 dermoscopic image samples, each of which has been annotated by clinical experts using a semi-automated or manual process similar to the ISIC 2017. In the pre-processing section, we randomly split the dataset into three sets: a training set with 1815 samples, a validation set with 259 samples, and a testing set with 520 samples. Similar to the ISIC 2017 pre-processing stage, to reduce network load and speed up the network training process, we resized all images’ spatial dimensions from 2016 × 3024 pixels to 256 × 256 pixels. Table 2 presents the comparison results of the proposed method against the SOTA approaches. The results indicate that our model outperformed the seven previous works based-on DSC and SP metrics.

**Figure 4:**
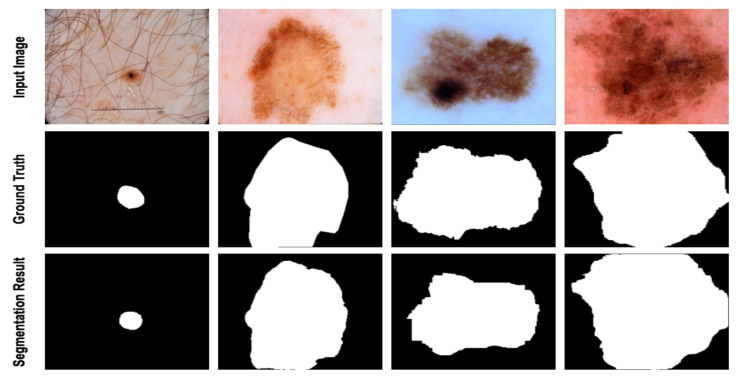
Segmentation results of the proposed method on ISIC 2018. The visualization shows that the proposed method learns the complex pattern of the lesion and precisely segments the abnormal regions.

To further analyze the segmentation performance of the proposed method, we provide Figure 4 to illustrate some segmentation maps obtained by our proposed network. It can be observed that the generated segmentation masks are quite precise in both object detection and boundary separation from the background.

#### 4.2.3. PH^2^ Dataset

The PH^2^ is another popular dataset in the field of skin lesion analysis, prepared by the dermatology services of Pedro Hispano Hospital, Matosinhos, Portugal. This dataset includes 200 dermoscopic images of skin lesions region that are gathered for future research on the classification and segmentation of cancerous regions in dermoscopic images. In the pre-processing stage, we followed the same procedure of a previous work [56] and randomly split the dataset into two subsets of 100 samples as a training set and 100 samples as a validation and a testing set.

**Figure 5:**
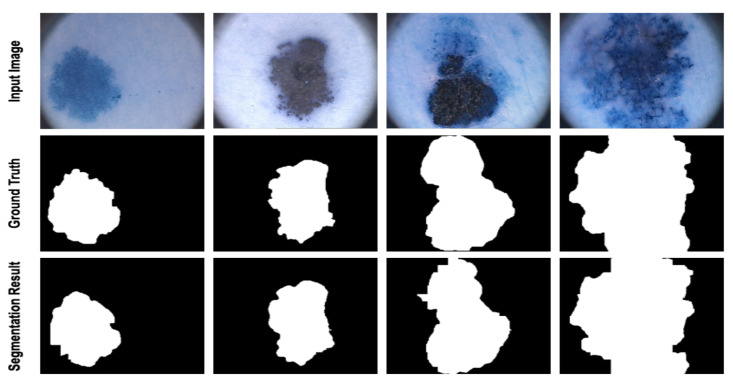
Segmentation results of the proposed method on PH^2^. The segmentation results illustrate that the proposed method accurately segmented the skin lesion area from the surrounding tissue region.

To validate the performance of the proposed method, we have provided Table 3 to quantitatively compare the obtained results with the SOTA approaches. Our results suggest that the proposed approach outperforms the SOTA methods in all metrics, excluding the FAT-Metusing SE metric. To illustrate the effectiveness of our network on segmenting skin lesion, we provide some visual examples of the segments generated by our network in Figure 5.

#### 4.2.4. Lung Segmentation Dataset

**Figure 6:**
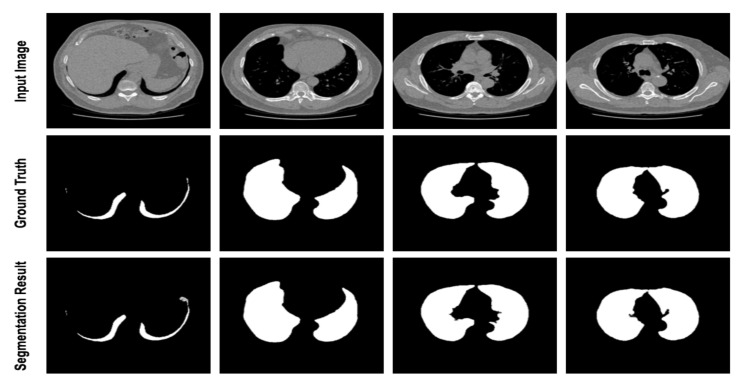
Segmentation results of the proposed method on lung dataset. The visualization shows that the proposed method learns the complex pattern of the lesion and precisely segments the abnormal regions.

This dataset is provided by the National Cancer Institute (NIH) and used in the Lung Nodule Analysis (LUNA) competition at the Kaggle Data Science Bowlin 2017 [64] to encourage researchers and scientists to develop lung cancer detection algorithms. This dataset includes lung computerized tomography (CT) scan images provided with annotations for lung segmentation. In Table 4, we have provided the comparison results of the proposed method against the counterparts to quantitatively analyze the obtained results. As it can be seen from the table that the suggested network slightly outperformed that the SOTA approaches in all metrics, except for the R2U-net and MCGU-Net with SE and SP metrics, respectively. We have also provided Figure 6 to visualize some segmentation results on the 2D scan of the lung dataset.

### 4.3. Ablation Study

To analyze the proposed structure in more detail, we conducted an ablation study. Throughout the ablation study, we used the ISIC 2018 dataset. To begin, we defined the baseline model as a seminal U-Net model without incorporating any of the proposed modules. Next, by inserting a transformer path we created the two-stream network where the CNN module learns local semantic representation while the Transformer module tries to encode the long-range contextual dependency. The resulting features of these two-streams were then fused using a concatenation operation followed by the convolutional layer. In the third setting, we replaced the concatenation operation with a one-directional ConvLSTM. For the last setting, we used the bi-directional ConvLSTM module to learn rich and generic representations. Results are presented in Table 5. It can be noticed that, by inserting each of the proposed modules, the entire model performance steadily improves and reaches the highest performance (in our experiments) using the combination of CNN+Transformer+Bi-directional ConvLSTM modules. It should also be noted that while these modules increase the performance at the same time they also increase the number of parameters to be trained. Thus, there is a trade-off between performance and computation complexity.

### 4.4. Discussion

In this work, we compared the performance of our suggested network with the SOTA approaches, e.g., MCGUnet, Fatnet and MedT. As the main dataset, we used three skin lesion segmentations, which contained diverse and challenging samples. As can be seen from Figure 3, Figure 4 and Figure 5, the annotation provided by the dermatologist (ground-truth mask) already contains the noisy labelling in the object boundary. Hence, boundary segmentation always comes with uncertainty. Our predicted results comparatively produce a better segmentation mask than the original noisy annotation and indeed it reveals the effectiveness of our approach in precise boundary separation. This fact might explain the importance of both local semantic and global dependency features for addressing such noise in the annotation mask.

## 5. Conclusions

This paper proposes a two-stream network where in the first stream a CNN module is incorporated to model the local semantic representation while the second stream utilizes a Transformer module to model long-range contextual dependency. To adaptively combine the generated features, it further applies a bi-directional ConvLSTM module to model both local and global interactions. Throughout the several experimental results on skin lesion segmentation datasets with overall accuracy ISIC 2017: 0.957, ISIC 2018: 0.949 and PH${}^{2}$: 0.971, on ISIC 2017 we have demonstrated that the proposed architecture is highly capable of learning rich and generic representation, which is crucial for the segmentation task. Furthermore, the experimental results on the lung segmentation dataset with an overall accuracy of 0.997 show that our method is extendable to other medical segmentation tasks. It should be noted that one drawback of our approach is the high number of parameters, hence, for a clinical application, future research should consider a parameter reduction technique in order to deploy the model in real-world scenarios.

## Figures and Tables

**Table 1:** Comparison results of the proposed method against the SOTA approaches on the ISIC 2017 dataset.

Method	DSC	SE	SP	ACC
U-Net [38]	0.8159	0.8172	0.9680	0.9164
Att U-Net [57]	0.8082	0.7998	0.9776	0.9145
DAGAN [58]	0.8425	0.8363	0.9716	0.9304
TransUNet [16]	0.8123	0.8263	0.9577	0.9207
MCGU-Net [56]	0.8927	0.8502	0.9855	0.9570
MedT [15]	0.8037	0.8064	0.9546	0.9090
FAT-Net [59]	0.8500	0.8392	0.9725	0.9326
ResU-Net [60]	0.8580	0.8492	0.9625	0.9403
ResU-Net++ [61]	0.8296	**0.8611**	0.9431	0.9228
SwinU-Net [62]	0.8866	0.8321	0.9738	0.9498
**Proposed Method**	**0.8929**	0.8482	**0.9819**	**0.9572**

**Table 2:** Comparison results of the proposed method against the SOTA approaches on the ISIC 2018 dataset.

Method	DSC	SE	SP	ACC
U-Net [38]	0.8545	0.8800	0.9697	0.9404
Att U-Net [57]	0.8566	0.8674	0.9863	0.9376
DAGAN [58]	0.8807	0.9072	0.9588	0.9324
TransUNet [16]	0.8499	0.8578	0.9653	0.9452
MCGU-Net [56]	0.895	0.8480	0.9860	0.9550
MedT [15]	0.8389	0.8252	0.9637	0.9358
FAT-Net [59]	0.8903	**0.9100**	0.9699	**0.9578**
SwinU-Net [62]	0.8916	0.8721	0.9632	0.9468
**Proposed Method**	**0.8932**	0.8932	**0.9800**	0.9490

**Table 3:** Comparison results of the proposed method against the SOTA approaches on the PH${}^{2}$ dataset.

Method	DSC	SE	SP	ACC
U-Net [38]	0.8936	0.9125	0.9588	0.9233
Att U-Net [57]	0.9003	0.9205	0.9640	0.9276
DAGAN [58]	0.9201	0.8320	0.9640	0.9425
TransUNet [16]	0.8840	0.9063	0.9427	0.9200
MCGU-Net [56]	0.9263	0.8322	0.9714	0.9537
MedT [15]	0.9122	0.8472	0.9657	0.9416
FAT-Net [59]	0.9440	**0.9441**	0.9741	0.9703
Atrous CNN [63]	0.7080	0.9041	0.9341	0.9003
SwinU-Net [62]	0.9322	0.9121	0.9711	0.9685
**Proposed Method**	**0.9442**	0.9233	**0.9750**	**0.9718**

**Table 4:** Comparison results of the proposed method against the SOTA approaches on the lung dataset.

Method	DSC	SE	SP	ACC
U-Net [38]	0.9658	0.9696	0.9872	0.9872
RU-net [65]	0.9638	0.9734	0.9866	0.9836
R2U-net [65]	0.9832	**0.9944**	0.9832	0.9918
MCGU-Net [56]	0.9904	0.9910	**0.9982**	0.9972
FRCU-Net [12]	0.9901	0.9904	0.9982	0.9970
**Proposed Method**	**0.9906**	0.9935	0.9967	**0.9973**

**Table 5:** Contribution of each of the proposed modules on the model performance.

Method	DSC	AC
**Baseline**	0.8545	0.9404
**Baseline + Transformer**	0.8664	0.9474
**Baseline + Transformer + ConvLSTM**	0.8863	0.9467
**proposed method**	**0.8932**	**0.9490**

## Data Availability

Not applicable.

## References

[1] Sharma M.K., Dhiman N., Mishra L., Mishra V., Sahani K. (2021). Mediative Fuzzy Extension Technique and Its Consistent Measurement in the Decision Making of Medical Application. Math. Probl. Eng..

[2] Dhiman N., Gupta M.M., Singh D.P., Mishra V.N., Sharma M.K. (2022). On Z-Intuitionistic Fuzzy Fractional Valuations for Medical Diagnosis: An Intuitionistic Fuzzy Knowledge-Based Expert System. Fractal Fract..

[3] Siegel R.L., Miller K.D., Jemal A. (2018). Cancer statistics, 2018. CA Cancer J. Clin..

[4] Ridge C.A., McErlean A.M., Ginsberg M.S. (2013). Epidemiology of lung cancer. Seminars in Interventional Radiology.

[5] Chen L.C., Papandreou G., Kokkinos I., Murphy K., Yuille A.L. (2017). Deeplab: Semantic image segmentation with deep convolutional nets, atrous convolution, and fully connected crfs. IEEE Trans. Pattern Anal. Mach. Intell..

[6] Li M., Lian F., Wang C., Guo S. (2021). Accurate pancreas segmentation using multi-level pyramidal pooling residual U-Net with adversarial mechanism. BMC Med. Imaging.

[7] Wang X., Girshick R., Gupta A., He K. Non-local neural networks. Proceedings of the IEEE Conference on Computer Vision and Pattern Recognition.

[8] Sinha A., Dolz J. (2020). Multi-scale self-guided attention for medical image segmentation. IEEE J. Biomed. Health Inform..

[9] Cai Y., Wang Y. (2020). Ma-unet: An improved version of unet based on multi-scale and attention mechanism for medical image segmentation. arXiv.

[10] Alahmadi M. (2022). Multi-scale Attention U-Net for Skin Lesion Segmentation. IEEE Access.

[11] Alahmadi M. (2022). Texture Attention Network for Diabetic Retinopathy Classification. IEEE Access.

[12] Azad R., Bozorgpour A., Asadi-Aghbolaghi M., Merhof D., Escalera S. Deep Frequency Re-calibration U-Net for Medical Image Segmentation. Proceedings of the IEEE/CVF International Conference on Computer Vision.

[13] Dosovitskiy A., Beyer L., Kolesnikov A., Weissenborn D., Zhai X., Unterthiner T., Dehghani M., Minderer M., Heigold G., Gelly S. (2020). An image is worth 16x16 words: Transformers for image recognition at scale. arXiv.

[14] Chen C.F.R., Fan Q., Panda R. Crossvit: Cross-attention multi-scale vision transformer for image classification. Proceedings of the IEEE/CVF International Conference on Computer Vision.

[15] Valanarasu J.M.J., Oza P., Hacihaliloglu I., Patel V.M. Medical transformer: Gated axial-attention for medical image segmentation. Proceedings of the International Conference on Medical Image Computing and Computer-Assisted Intervention.

[16] Chen J., Lu Y., Yu Q., Luo X., Adeli E., Wang Y., Lu L., Yuille A.L., Zhou Y. (2021). Transunet: Transformers make strong encoders for medical image segmentation. arXiv.

[17] Hatamizadeh A., Tang Y., Nath V., Yang D., Myronenko A., Landman B., Roth H.R., Xu D. Unetr: Transformers for 3d medical image segmentation. Proceedings of the IEEE/CVF Winter Conference on Applications of Computer Vision.

[18] Garcia-Arroyo J.L., Garcia-Zapirain B. (2019). Segmentation of skin lesions in dermoscopy images using fuzzy classification of pixels and histogram thresholding. Comput. Methods Programs Biomed..

[19] Pereira P.M., Tavora L.M., Fonseca-Pinto R., Paiva R.P., Assunção P.A.A., de Faria S.M. Image Segmentation using Gradient-based Histogram Thresholding for Skin Lesion Delineation. Proceedings of the 12th International Joint Conference on Biomedical Engineering Systems and Technologies (BIOSTEC 2019).

[20] Yueksel M.E., Borlu M. (2009). Accurate segmentation of dermoscopic images by image thresholding based on type-2 fuzzy logic. IEEE Trans. Fuzzy Syst..

[21] Kockara S., Mete M., Yip V., Lee B., Aydin K. (2010). A soft kinetic data structure for lesion border detection. Bioinformatics.

[22] Ashour A.S., Hawas A.R., Guo Y., Wahba M.A. (2018). A novel optimized neutrosophic k-means using genetic algorithm for skin lesion detection in dermoscopy images. Signal Image Video Process..

[23] Azad R., Ahmadzadeh E., Azad B. (2015). Real-time human face detection in noisy images based on skin color fusion model and eye detection. Intelligent Computing, Communication and Devices.

[24] Wong A., Scharcanski J., Fieguth P. (2011). Automatic skin lesion segmentation via iterative stochastic region merging. IEEE Trans. Inf. Technol. Biomed..

[25] Salih O., Viriri S. (2020). Skin lesion segmentation using stochastic region-merging and pixel-based Markov random field. Symmetry.

[26] Emre Celebi M., Kingravi H.A., Iyatomi H., Alp Aslandogan Y., Stoecker W.V., Moss R.H., Malters J.M., Grichnik J.M., Marghoob A.A., Rabinovitz H.S. (2008). Border detection in dermoscopy images using statistical region merging. Skin Res. Technol..

[27] Riaz F., Naeem S., Nawaz R., Coimbra M. (2018). Active contours based segmentation and lesion periphery analysis for characterization of skin lesions in dermoscopy images. IEEE J. Biomed. Health Inform..

[28] Tang J. (2009). A multi-direction GVF snake for the segmentation of skin cancer images. Pattern Recognit..

[29] Silveira M., Nascimento J.C., Marques J.S., Marçal A.R., Mendonça T., Yamauchi S., Maeda J., Rozeira J. (2009). Comparison of segmentation methods for melanoma diagnosis in dermoscopy images. IEEE J. Sel. Top. Signal Process..

[30] Ali A.R., Couceiro M.S., Hassenian A.E. Melanoma detection using fuzzy C-means clustering coupled with mathematical morphology. Proceedings of the 2014 14th International Conference on Hybrid Intelligent Systems.

[31] Burdick J., Marques O., Weinthal J., Furht B. (2018). Rethinking skin lesion segmentation in a convolutional classifier. J. Digit. Imaging.

[32] Zhang X., Thibault G., Decencière E., Marcotegui B., Laÿ B., Danno R., Cazuguel G., Quellec G., Lamard M., Massin P. (2014). Exudate detection in color retinal images for mass screening of diabetic retinopathy. Med. Image Anal..

[33] Fraz M.M., Barman S.A., Remagnino P., Hoppe A., Basit A., Uyyanonvara B., Rudnicka A.R., Owen C.G. (2012). An approach to localize the retinal blood vessels using bit planes and centerline detection. Comput. Methods Programs Biomed..

[34] Lam B.S., Gao Y., Liew A.W.C. (2010). General retinal vessel segmentation using regularization-based multiconcavity modeling. IEEE Trans. Med. Imaging.

[35] Hu S., Hoffman E.A., Reinhardt J.M. (2001). Automatic lung segmentation for accurate quantitation of volumetric X-ray CT images. IEEE Trans. Med. Imaging.

[36] Mansoor A., Bagci U., Xu Z., Foster B., Olivier K.N., Elinoff J.M., Suffredini A.F., Udupa J.K., Mollura D.J. (2014). A generic approach to pathological lung segmentation. IEEE Trans. Med. Imaging.

[37] Long J., Shelhamer E., Darrell T. Fully convolutional networks for semantic segmentation. Proceedings of the IEEE Conference on Computer Vision and Pattern Recognition.

[38] Ronneberger O., Fischer P., Brox T. U-net: Convolutional networks for biomedical image segmentation. Proceedings of the International Conference on Medical Image Computing and Computer-Assisted Intervention.

[39] Çiçek Ö., Abdulkadir A., Lienkamp S.S., Brox T., Ronneberger O. 3D U-Net: Learning dense volumetric segmentation from sparse annotation. Proceedings of the International Conference on Medical Image Computing and Computer-Assisted Intervention.

[40] Zhou Z., Rahman Siddiquee M.M., Tajbakhsh N., Liang J. (2018). Unet++: A nested u-net architecture for medical image segmentation. Deep Learning in Medical Image Analysis and Multimodal Learning for Clinical Decision Support.

[41] Badrinarayanan V., Kendall A., Cipolla R. (2017). Segnet: A deep convolutional encoder-decoder architecture for image segmentation. IEEE Trans. Pattern Anal. Mach. Intell..

[42] Azad R., Rouhier L., Cohen-Adad J. Stacked Hourglass Network with a Multi-level Attention Mechanism: Where to Look for Intervertebral Disc Labeling. Proceedings of the International Workshop on Machine Learning in Medical Imaging.

[43] Liu L., Tsui Y.Y., Mandal M. (2021). Skin lesion segmentation using deep learning with auxiliary task. J. Imaging.

[44] Tong X., Wei J., Sun B., Su S., Zuo Z., Wu P. (2021). ASCU-Net: Attention gate, spatial and channel attention u-net for skin lesion segmentation. Diagnostics.

[45] Kim Y.G., Kim K., Wu D., Ren H., Tak W.Y., Park S.Y., Lee Y.R., Kang M.K., Park J.G., Kim B.S. (2022). Deep learning-based four-region lung segmentation in chest radiography for COVID-19 diagnosis. Diagnostics.

[46] Liu L., Huang Z., Liò P., Schönlieb C.B., Aviles-Rivero A.I. (2022). PC-SwinMorph: Patch Representation for Unsupervised Medical Image Registration and Segmentation. arXiv.

[47] Meng X., Zhang X., Wang G., Zhang Y., Shi X., Dai H., Wang Z., Wang X. (2021). Exploiting full Resolution Feature Context for Liver Tumor and Vessel Segmentation via Fusion Encoder: Application to Liver Tumor and Vessel 3D reconstruction. arXiv.

[48] Xu G., Wu X., Zhang X., He X. (2021). Levit-unet: Make faster encoders with transformer for medical image segmentation. arXiv.

[49] Chollet F. Xception: Deep learning with depthwise separable convolutions. Proceedings of the IEEE Conference on Computer Vision and Pattern Recognition.

[50] Shi X., Chen Z., Wang H., Yeung D.Y., Wong W.K., Woo W.C. (2015). Convolutional LSTM network: A machine learning approach for precipitation nowcasting. Adv. Neural Inf. Process. Syst..

[51] Song H., Wang W., Zhao S., Shen J., Lam K.M. Pyramid dilated deeper convlstm for video salient object detection. Proceedings of the European Conference on Computer Vision (ECCV).

[52] Cui Z., Ke R., Pu Z., Wang Y. (2018). Deep bidirectional and unidirectional LSTM recurrent neural network for network-wide traffic speed prediction. arXiv.

[53] Codella N.C., Gutman D., Celebi M.E., Helba B., Marchetti M.A., Dusza S.W., Kalloo A., Liopyris K., Mishra N., Kittler H. Skin lesion analysis toward melanoma detection: A challenge at the 2017 international symposium on biomedical imaging (isbi), hosted by the international skin imaging collaboration (isic). Proceedings of the 2018 IEEE 15th International Symposium on Biomedical Imaging (ISBI 2018).

[54] Codella N., Rotemberg V., Tschandl P., Celebi M.E., Dusza S., Gutman D., Helba B., Kalloo A., Liopyris K., Marchetti M. (2019). Skin lesion analysis toward melanoma detection 2018: A challenge hosted by the international skin imaging collaboration (isic). arXiv.

[55] Mendonça T., Ferreira P.M., Marques J.S., Marcal A.R., Rozeira J. PH 2-A dermoscopic image database for research and benchmarking. Proceedings of the 2013 35th Annual International Conference of the IEEE Engineering in Medicine and Biology Society (EMBC).

[56] Asadi-Aghbolaghi M., Azad R., Fathy M., Escalera S. (2020). Multi-level context gating of embedded collective knowledge for medical image segmentation. arXiv.

[57] Oktay O., Schlemper J., Folgoc L.L., Lee M., Heinrich M., Misawa K., Mori K., McDonagh S., Hammerla N.Y., Kainz B. (2018). Attention u-net: Learning where to look for the pancreas. arXiv.

[58] Lei B., Xia Z., Jiang F., Jiang X., Ge Z., Xu Y., Qin J., Chen S., Wang T., Wang S. (2020). Skin lesion segmentation via generative adversarial networks with dual discriminators. Med. Image Anal..

[59] Wu H., Chen S., Chen G., Wang W., Lei B., Wen Z. (2022). FAT-Net: Feature adaptive transformers for automated skin lesion segmentation. Med. Image Anal..

[60] Zafar K., Gilani S.O., Waris A., Ahmed A., Jamil M., Khan M.N., Sohail Kashif A. (2020). Skin lesion segmentation from dermoscopic images using convolutional neural network. Sensors.

[61] Ashraf H., Waris A., Ghafoor M.F., Gilani S.O., Niazi I.K. (2022). Melanoma segmentation using deep learning with test-time augmentations and conditional random fields. Sci. Rep..

[62] Cao H., Wang Y., Chen J., Jiang D., Zhang X., Tian Q., Wang M. (2021). Swin-unet: Unet-like pure transformer for medical image segmentation. arXiv.

[63] Kaur R., GholamHosseini H., Sinha R., Lindén M. (2022). Automatic lesion segmentation using atrous convolutional deep neural networks in dermoscopic skin cancer images. BMC Med. Imaging.

[64] (2022). Finding and Measuring Lungs in CT Data. https://www.kaggle.com/datasets/kmader/finding-lungs-in-ct-data.

[65] Alom M.Z., Hasan M., Yakopcic C., Taha T.M., Asari V.K. (2018). Recurrent residual convolutional neural network based on u-net (r2u-net) for medical image segmentation. arXiv.

